# Chitinases as Food Allergens

**DOI:** 10.3390/molecules24112087

**Published:** 2019-05-31

**Authors:** Claudia Leoni, Mariateresa Volpicella, Maria C.G. Dileo, Bruno A.R. Gattulli, Luigi R. Ceci

**Affiliations:** 1 Institute of Bioenergetics, Biomembranes and Molecular Biotechnologies, Italian National Research Council, 70126 Bari, Italy; c.leoni@ibiom.cnr.it (C.L.); b.gattulli@ibiom.cnr.it (B.A.R.G.); 2 Department of Biosciences, Biotechnologies and Biopharmaceutics Sciences, University of Bari, 70126 Bari, Italy; 3 Department of Biology, University of Bari, 70126 Bari, Italy; mariacristina.g.1993@gmail.com

**Keywords:** chitinase, allergen, food allergy, epitope mapping

## Abstract

Food allergies originate from adverse immune reactions to some food components. Ingestion of food allergens can cause effects of varying severity, from mild itching to severe anaphylaxis reactions. Currently there are no clues to predict the allergenic potency of a molecule, nor are cures for food allergies available. Cutting-edge research on allergens is aimed at increasing information on their diffusion and understanding structure-allergenicity relationships. In this context, purified recombinant allergens are valuable tools for advances in the diagnostic and immunotherapeutic fields. Chitinases are a group of allergens often found in plant fruits, but also identified in edible insects. They are classified into different families and classes for which structural analyses and identification of epitopes have been only partially carried out. Moreover, also their presence in common allergen databases is not complete. In this review we provide a summary of the identified food allergenic chitinases, their main structural characteristics, and a clear division in the different classes.

## 1. Introduction

Food allergies originating from a cross-link reaction between specific food proteins (the allergens) and IgEs (immunoglobulin Es) represent a serious health concern in everyday life, also due to the increasing habits to consume ready-to-eat products. In Western countries, it is estimated that food allergies affect about 5% of adults and 8% of children [1]. Also, in Eastern countries the problem is emerging and is being addressed with growing interest [2,3,4].

The symptoms of IgE-mediated allergies can be various, including mild pruritus and even life-threatening anaphylaxis reactions. Currently there is no cure for food allergy, and allergic subjects are compelled to extreme avoidance of allergenic (and suspect) foods. Therefore, studies on the identification and characterization of single food allergens (and their genes) are of main interest to understand their pathogenicity, their diffusion within food sources, the structural basis of their allergenicity and the possibility of their adoption in molecular-based protocols for allergy diagnosis, therapy and prevention [5,6]. Accordingly, molecular studies, including gene cloning and expression of recombinant proteins, directed-evolution analysis, protein purification and structure determination, bioinformatics and protein structure prediction, are gaining momentum in advanced allergology [7,8,9].

Food allergens belong to a limited number of protein families [5,7] (see also the AllFam database at http://www.meduniwien.ac.at/allfam/) and at the moment there is no clue about the structural characteristics underlying allergenicity. This review focuses on the identification, molecular characterization and analysis of food allergenic chitinases. It represents an update and integration of a previous review on plant chitinases as food allergens [10]. Chitinases are a well-known group of food allergens, mainly of plant origins. Even if many of them have been detected in fresh fruits and berries, and therefore not subjected to any food processing before assumption, it also emerged that chitinases present in processed food (flour, beer and polenta) can be allergenic. Furthermore, taking into consideration the physiological role of chitinases as pathogenesis-related proteins, their possible overexpression in plants attacked by pathogens can constitute an additional risk for allergic subjects [11].

Chitinases (EC 3.2.1.14) catalyze the hydrolysis of the β-1,4-N-acetyl-D-glucosamine linkages in chitin polymers. The enzyme is produced by a wide range of organisms including bacteria, fungi, insects, plants and vertebrates for different purposes such as nutrition, morphogenesis and defence against chitin-containing pathogens [12]. A complete list of chitinases can be found in the Carbohydrate-Active enZYmes database (CAZY) website, http://www.cazy.org/Glycoside-Hydrolases.html, in which they are classified within the families 18 and 19 of glycoside hydrolases (GH), according to their primary sequences. Members of the two families also differ in the stereochemistry of the hydrolysis: while GH18 chitinases act according to a retaining mechanism of the anomeric substrate, GH19 members react by means of an inverting mechanism. The GH18 family comprises about 17,800 chitinases, including about 13,600 of bacterial origin, 3500 from eukaryotes and 300 from viruses. They are characterized by a signal peptide and a conserved (β/α)_8_ structure containing the catalytic module. The GH19 family is composed by about 7000 enzymes mainly from Bacteria (5000), Eukaryotes (1000) and Viruses (900). Their 3D structure is not defined.

Plant chitinases are also distinguished in several classes according to different lengths and architectures. Some GH19 chitinases contain a chitin-binding domain located just after the signal peptide and are further distinguished between class I and class IV, mainly on the basis of the length of the catalytic module, which is about 50 amino acids shorter in those of class IV. GH19 chitinases which do not contain the chitin binding domain are classified within class II. The GH18 chitinases are further classified in two different classes (class III and V), both not provided with the chitin binding module [13].

An allergenic chitinase was also described in a type of silkworm pupae (*Bombyx mori L.*) used for food [14]. It is the first food allergenic chitinase outside of plants. Its detection deserves particular interest since silkworm pupae are a traditional food in East Asia, which can be of possible development as a new food habit in other countries.

For both plant and insect chitinases the possibility that the allergic reaction is due to the so-called cross-reactivity syndrome, i.e., the onset of food allergy in subjects already exposed and sensitized to structurally similar non food allergens, can be considered. In particular, for many plant chitinases a structural similarity with hevein (a protein identified as a major allergen for patients allergic to the latex extracted from the rubber tree *Hevea brasiliensis* [15]) has been reported since the first descriptions of allergenic chitinases in plants [16,17]. Also, for the recently identified insect allergenic chitinase, a structural similarity with an allergenic chitinase from the house dust mite *Dermatophagoides farinae* (Der f 18) has been reported [14].

The chitinases identified as possible food allergens are reported in Table 1. For each allergen its origin (Source) and the type of molecule identified were reported. In particular, in the Molecule column, I stands for allergens identified on the basis of partial sequencing (or mass spectrometry analysis); P indicates allergens purified from the food source; R corresponds to allergens available as recombinant proteins. Regarding the allergenicity assay (Assay column), IR indicates the allergens identified in food extracts, or tested as a single protein, by immunoblot reaction with sera of allergic patients. SPT represents the skin prick test sometimes used to assay the allergenicity of pure proteins. The IUIS, UniProt and PDB columns show the respective accession numbers (if available).

Different classes of allergenic chitinases are also briefly introduced. Specific attention is given to the description, when available, of the possible epitopes of the different classes, since their identification and mapping constitute a main objective in the study of allergens by molecular approaches. Data obtained for new allergenic chitinases are also considered to clarify which protein domain is responsible for allergenicity. Indeed, the classical view that allergenicity is caused by the so-called chitin-binding module, according to the high similarity of this region with the allergenic hevein, should now be reviewed, due to the identification of several allergenic chitinases lacking this domain.

## 2. Class I

This class contains thirteen allergenic chitinases, identified in foods of plant origin (Table 1). They are molecules of about 30 KDa deriving from polypeptides of 315–332 amino acids containing a signal peptide of 18–26 amino acids. Downstream of the signal peptide a chitin-binding module of 41–42 amino acids is present. It results highly folded thanks to the presence of 4 S-S bridges.

Recently an allergenic chitinase from rice has been described as a member of this class based on the high sequence identities with the class I chitinases from *H. brasiliensis*, *C. sativa* and *P. americana* [28]. However, we believe that it should be better classified within the class II chitinases. In fact, the allergen was produced as recombinant molecule starting from a cDNA sequence encoding only the catalytic module, and therefore there is no evidence of a protein structure corresponding to the class I chitinases. Furthermore, its sequence identities with the class II chitinases are higher than those with chitinases of class I (See Supplementary Material Table S1). Interestingly, however, this chitinase (even if not provided of a chitin binding module) was found to be allergenic, giving further support to the possible role of the catalytic module in the allergenicity of chitinases (for further details see the Class II paragraph).

**Figure 1:**
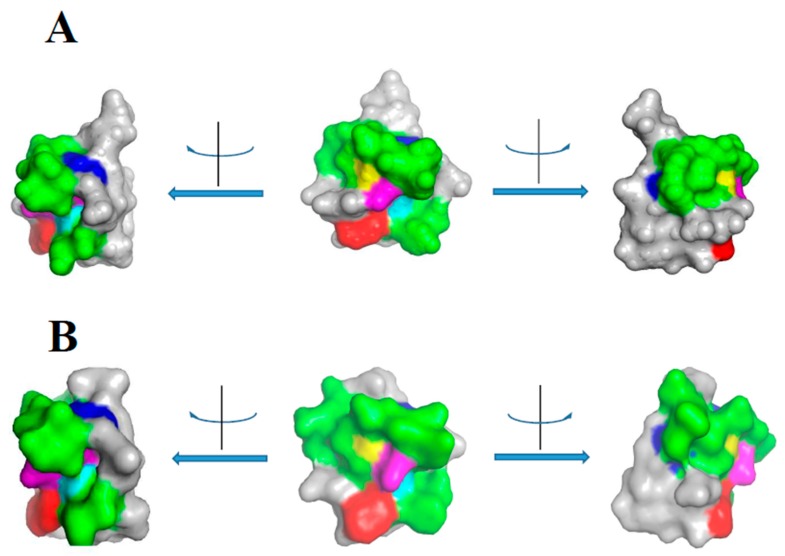
Epitope mapping on hevein and class I chitinases. (**A**) Epitope mapping on hevein 3D structure. The reported structure was obtained from the 3D structure of the protein (PDB 4MPI) by restriction to Chain A and by selection of the amino acids 12–31, corresponding to the consensus sequence C_12_XXXXCCSXAφXφCGXΩXAcYC_31_. Colour code: Blue, C12 and C31; cyan, S19; red, W23; yellow, G25; magenta, Y30. The molecule is shown from three different points of view, corresponding to clockwise and counterclockwise rotations of 90°. (**B**) Epitope mapping on the 3D model of the chestnut chitinase. The model was obtained by supplying the sequence of the chestnut chitin binding module to the Phyre2 program [39] using the hevein structure (4MPI) as template. Coloured amino acids are as for the hevein structure. All images were prepared by using PyMol [40] resources.

In Figure S1 the multialignment of chitinases of this class I is shown. Similarities among chitinase binding modules and among catalytic modules are about 60% and 65%, respectively. Currently there are no studies on the identification of possible epitopes of these chitinases. Epitopes were identified only for the structurally similar hevein, thanks to immune-reactivity analysis of both mutant [37] and synthetic peptides [38]. Overall, these studies allowed to identify a consensus motif C_12_XXXXCCSXφXφCGXΩXAcYC_31_ (X = any amino acid; φ = an aromatic amino acid; Ω = T, S or G; Ac = E or D) which can also be detected in chitinases of class I and IV. Figure 1A reports the 3D structure of hevein (PDB 4MPI) in which the consensus sequence is highlighted. It results almost entirely on the molecule surface. For comparison, the same region was highlighted on the 3D model of the chestnut chitin binding module (amino acids 19–59 of the Q42428 sequence) (Figure 1B).

## 3. Class II

In this class, five plant allergenic chitinases can be classified. Molecules of this class lack the chitin binding module and their catalytic modules show an identity of about 70% with the corresponding region of the class I chitinases. They have a molecular weight of about 25 KDa and contain between 2–4 disulphide bridges. The identification in tomato berries of two allergenic chitinases must alert those who consume the raw product.

**Figure 2:**
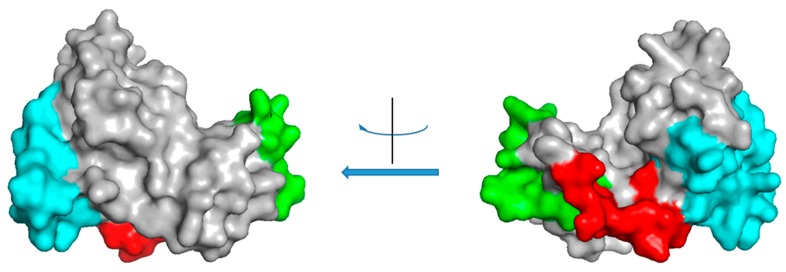
Epitope mapping on the predicted 3D structure of a class II rice chitinase. The reported 3D structure was obtained for the rice chitinase protein (UniProt O24007) by Phyre2 prediction. The program predicted a structure with 100% confidence on the template 2DKV, a rice chitinase with 62% identity with the query sequence. Coloured regions correspond to predicted linear epitope described by Mishra et al. [28]. Colour code: red, epitope E_12_TTGGTRGSSDQFQ_25_; green, epitope K_31_EEINKATSPPYYGR_45_; cyan, epitope N_140_DANVDRIGYYKRYCDMLGTGYGSNLD_166_. The molecule is shown from two different points of view, corresponding to a rotation of 180°.

The latest chitinase described in this class was identified in rice. Its allergenicity was recognized both by using predictive bioinformatic approaches and by ELISA immunogenicity assays conducted on the recombinant molecule using sera of seven allergic subjects [28]. This study represents the first case of a recombinant class II chitinase analysed by immunogenic assays. Interestingly the protein was found reactive in western blot analysis even after heat treatment at 90 °C for 60 min, supporting the potential allergenicity of chitinases in processed foods. By bioinformatic analysis, possible linear and conformational epitopes were identified. Two synthetic peptides corresponding to putative epitopes were also assayed by ELISA assays. The three identified epitopes are shown on the predicted rice chitinase 3D structure in Figure 2. The alignment of the class II chitinases with the rice chitinase is reported in Figure S2.

## 4. Class III

This class of allergenic chitinases contains four plant chitinases of about 25–30 KDa. Complete information for gene sequences is available only for the two molecules from Indian jujube and pomegranate. Also these proteins are encoded with a presequence of 24 and 26 amino acids, respectively.

**Figure 3:**
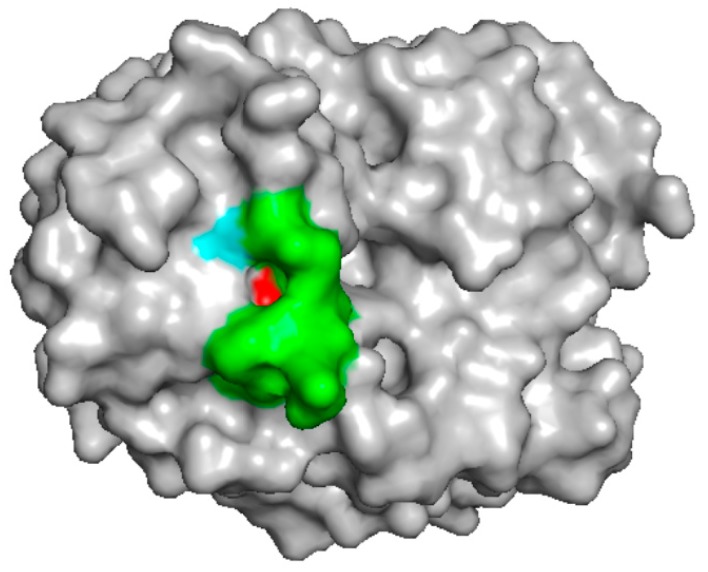
Epitope mapping on the 3D structure of the class III pomegranate chitinase (PDB 4TOQ). The region P_221_AAPEAAGSG_230_ of the pomegranate chitinase, resulting 80% identical to an epitope described in velvet grass [42], was coloured and shown in the protein 3D structure. The selected region was coloured green, except for the terminal amino acids: P_221_ (red) and G_230_ (cyan).

The chitinase from pomegranate was recently described [32]. It was identified by N-terminal sequencing and immunoblot analysis against sera of allergic patients. Its sequence corresponds to an already identified chitinase of pomegranate (UniProt G1UH28) whose 3D structure was determined by X-ray diffraction (PDB 4TOQ). The protein structure consists of ten β-strands and eight α-helices, of which β/α motifs form a TIM-barrel structure [41]. There are no studies on the identification of possible epitopes on chitinases of this class. Using the pomegranate sequence as query we performed a Blast epitope searching within the IEDB (Immune Epitope Database and Analysis Resource, https://www.iedb.org) [42] and identified a region with a similarity of 80% with the pollen allergen Hol I 5 from velvet grass (*Holcus lanatus*) [43]. This region (P_221_AAPEAAGSG_230_) results displayed on the surface of the pomegranate protein structure (Figure 3). It is also entirely conserved in the hevamine protein (UniProt P23472), but it is only partially shared by the other two food allergenic chitinases of this class (see Figure S3). More detailed studies, therefore, are necessary to identify epitopes of class III chitinases.

## 5. Class IV

Allergenic chitinases from grape and maize are included in this class. These molecules contain a chitin binding module and show a similarity of about 40–50% with the class I chitinases. Their chitin binding module, of 35–36 amino acids, is shorter than that of class I chitinases and contains a reduced number of disulphide bonds. In Figure S4 the multialignment of the identified plant class IV allergenic chitinases is reported.

One grape chitinase, including its chitin binding module, was expressed in insect cells [35]. It resulted allergenic, as demonstrated by immunoblot assay against the sera of patients allergic to grape.

**Figure 4:**
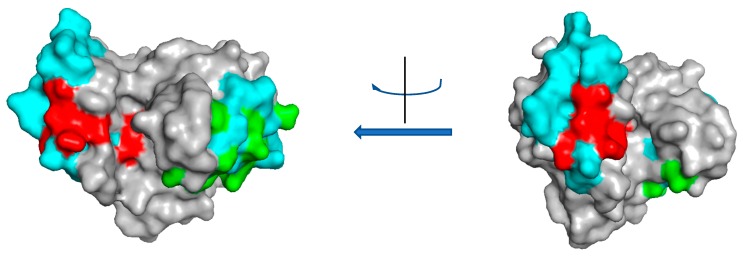
Epitope mapping on the 3D structure of the class IV maize chitinase (PDB 4MCK). The regions I_151_–R_179_ and N_252_–I_279_ of the maize chitinase corresponding to the epitopes K_31_EEINKATSPPYYGR_45_ and N_140_DANVDRIGYYKRYCDMLGTGYGSNLD_166_ of the rice class II chitinase [28] were selected and coloured in cyan. In addition, the amino acids of the two epitopes that are conserved between the rice and maize chitinases were coloured in red (for the first epitope) and green (for the second epitope). The molecule is shown from two different points of view, corresponding to a rotation of 90°.

Interestingly, the catalytic module of the maize allergenic chitinase was expressed as recombinant molecule in *E. coli* cells and resulted immunoreactive against sera of subjects allergic to maize, clearly demonstrating that the allergenicity of chitinases is not restricted to the chitin binding domain [36]. A comparison with the epitopes identified in the rice chitinase of class II reveals the presence of similar sequences in class IV chitinases (See Figure S4). In Figure 4, regions corresponding to two of the epitopes identified in the catalytic module of the allergenic rice class II chitinase have been highlighted in the 3D structure of the maize chitinase (PDB 4MCK). These are regions to be further investigated as possible epitopes in the maize chitinase. Nevertheless, the possibility that also the chitin binding module could result allergenic cannot be ruled out due to its high identity with the allergenic hevein (see Figure S4).

## 6. Insect Chitinases

A food allergenic chitinase was also identified in silkworm (*B. mori* L.) pupae [14]. Silkworm pupae are nutrient-rich food, regularly consumed in China, India and other countries with developed sericulture. Even if they are eaten fried or boiled, they can retain their allergenicity [44]. By immunoblot analysis of the pupae proteins with sera of four patients with severe allergy episodes after ingestion of silkworms, two main allergenic proteins were identified (a chitinase and a paramyosin). The chitinase was identified as a protein of 555 amino acids, including a signal peptide of 20 amino acids, belonging the GH18 family. It is the first description of an allergenic chitinase outside of plants and, taking into account the large consumption of insects as food in the Eastern Countries [45] and also in anticipation of their adoption as food in Western countries [46], it represents an important issue to be addressed for food safety. In this regard, it should be noted that also an arginine kinase was described as possible food allergen in *B. mori* [47].

The silkworm chitinase shares few traits with the plant counterparts. As a member of the GH18 family, it possesses the DXDXE motif including the catalytic Glu (D_306_IDWE_310_). The molecule is about 200 amino acids longer than plant chitinases, mainly for the presence of an extended N-terminal region. From a structural point of view, it resembles more a class V chitinase than a class III one. In fact, it has higher identities with class V chitinases (not dealt in this review, since no allergenic molecules have been described) and it is not provided with the six cysteines typical of class III chitinases. Currently, there are no studies on the identification of epitopes on this molecule.

## 7. Conclusions

Food allergenic chitinases are a relatively small group of proteins, but their relevance as allergens cannot be underestimated given their presence in highly consumed fruits and plant derivatives. It is therefore necessary to have a clear representation of their diffusion, allergenic potency and structural characteristics. We have reported an updated collection of all the allergenic chitinases identified so far in food, including information on their molecular analysis. Some interesting and not yet adequately considered aspects emerged, such as the first complete characterization of an allergen of the class II; the confirmation that the allergenicity of chitinases is not limited to the hevein-like chitin-binding module and the identification of the first allergenic chitinase outside of the plant kingdom. These findings indicate that further efforts are still needed to achieve a robust characterization of allergenic chitinases (and also of all the other allergens). In this regard, advances in genomics, bioinformatics and molecular biology technologies will undoubtedly make a strong contribution.

## Figures and Tables

**Table 1:** Chitinases identified as food allergens.

Source	Molecule	Assay	IUIS	UniProt	PDB	Ref	Note
**Class I (GH19, with chitin binding module)**							
Kiwi fruit (*Actidinia chinensis*)	I	IR				[18]	
Papaya (*Carica papaya*)	I					[19]	
Chestnut (*Castanea sativa*)	I		Cas s 5	Q42428			
Tomato (*Lycopersicum esculentum*)	I	IR		Q05538		[20]	
Banana fruits (*Musa sp*)	P	IR, SPT	Mus a 2	Q8VXF1, B6UYK6, C3VD22, O22318, M0SI55, M0SI56		[21,22,23]	
Avocado (*Persea americana*)	P,R	IR	Pers a I	P93680		[24,25]	
Green bean (*Phaseolus vulgaris*)	I	SPT		P36361		[26]	
Wheat (*Triticum aestivum*)	I	IR		Q6T484		[27]	
**Class II (GH19, without chitin binding module)**							
Tomato (*L. esculentum*)			Sola I	Q7Y0S1 Q05539			a
Wheat (*T. aestivum*)	I			Q4Z8L8 Q8W429		[27]	
Rice (*Oryza sativa*)	R	IR		O24007	Figure 2	[28]	
**Class III (GH18, without chitin binding module)**							
Coffee green beans (*Coffea arabica*)	I, R	IR	Cof a 1	D7REL9		[29]	
Raspberry berries (*Rubus ideaeu*)	I	IR				[30]	
Indian jujube fruit (*Zizyphus mauritiana*)	P,R	IR	Ziz m 1	Q2VST0		[31]	
Pomegranate (*Punica granatum*)	I	IR, SPT		G1UH28	4TOQ	[32]	
**Class IV (GH19, with chitin binding module)**							
Grape (*Vitis vinifera*)	I, R	IR		O2453, O24530		[33,34,35]	
Maize (*Zea mays*)	R	IR	Zea m 8	P29022	4MCK	[36]	
**Insect chitinases (GH18)**							
Silkworm (*Bombyx mori*)	I	IR		Q869E2		[14]	

In the Molecules column, I, P and R indicate allergens identified, purified or produced as recombinant molecule, respectively. In the assay column, IR and SPT refer to allergens assayed by immunoblot reactions or skin prick test. The web sites for the databases are the follow: IUIS: http://www.allergen.org/; UniProt: https://www.uniprot.org/; PDB: https://www.rcsb.org/. a) Sola I is only reported in the Allergome database. It is reported as a class I chitinase and is linked to Q05539 and Q7Y0S1 proteins. These molecules, however, do not show the chitin binding module and should be considered as class II chitinases.

## Supplementary Materials

The following are available online at https://www.mdpi.com/1420-3049/24/11/2087/s1, Table S1: Percent identity matrix obtained by Clustal2.1 for chitinases of class I and class II. Figure S1: Multiple alignment of Class I chitinases; Figure S2: Multiple alignment of Class II chitinases; Figure S3: Multiple alignment of Class III chitinases; Figure S4: Multiple alignment of Class IV chitinases.
