# Supplementary material for: Chitinases as Food Allergens
Source: Molecules. 2019 May 31;24(11):2087. doi: 10.3390/molecules24112087 (PMC6600546; doi:10.3390/molecules24112087)
Supplement: Supplementary file 1 [file molecules-24-02087-s001.pdf]

# Supplementary Materials

# Chitinases as food allergens

Claudia Leoni[1], Mariateresa Volpicella[2,*], Maria C.G. Dileo[3], Bruno A.R. Gattulli[1] and Luigi R. Ceci[1,*]

[1] Institute of Bioenergetics, Biomembranes and Molecular Biotechnologies, Italian National Research Council, Via Amendola 165/a, 70126 Bari (Italy);
[2] Department of Biosciences, Biotechnologies and Biopharmaceutic Sciences and
[3] Department of Biosciences University of Bari, Via Amendola 165/a, 70126 Bari (Italy).

* Authors for correspondence:
M. Volpicella, mariateresa.volpicella@uniba.it
L.R. Ceci, luigi.ceci@ibiom.cnr.it

Keywords: Chitinase; allergen; food allergy; epitope mapping

**List of Supplementary Materials:**
- **Table S1. Percent identity matrix obtained by Clustal2.1 for chitinases of class I and class II.**
- **Figure S1. Multiple alignment of Class I chitinases.**
- **Figure S2. Multiple alignment of Class II chitinases**.
- **Figure S3. Multiple alignment of Class III chitinases**.
- **Figure S4. Multiple alignment of Class IV chitinases.**

## Table S1

| | | | | | | | | |
|---|---|---|---|---|---|---|---|---|
| 1: Q7Y0S1 | 100.00 | 65.88 | 55.95 | 63.11 | 60.35 | 61.61 | 62.11 | 60.79 |
| 2: O24007 | 65.88 | 100.00 | 61.49 | 65.90 | 59.43 | 66.67 | 64.00 | 65.14 |
| 3: P36361 | 55.95 | 61.49 | 100.00 | 67.61 | 68.75 | 71.49 | 71.48 | 69.38 |
| 4: Q42428 | 63.11 | 65.90 | 67.61 | 100.00 | 70.04 | 71.78 | 75.00 | 72.18 |
| 5: Q05538 | 60.35 | 59.43 | 68.75 | 70.04 | 100.00 | 74.49 | 72.37 | 72.76 |
| 6: Q4Z818 | 61.61 | 66.67 | 71.49 | 71.78 | 74.49 | 100.00 | 75.72 | 73.66 |
| 7: Q8VXF1 | 62.11 | 64.00 | 71.48 | 75.00 | 72.37 | 75.72 | 100.00 | 78.68 |
| 8: P93680 | 60.79 | 65.14 | 69.38 | 72.18 | 72.76 | 73.66 | 78.68 | 100.00 |

**Table S1. Percent identity matrix obtained by Clustal2.1** [1] **for chitinases of class I and class II.** Only sequences corresponding to the catalytic module were aligned. Names of sequences are as reported in Table 1. Sequences highlighted in green belong to class II.

**Figure S1. Multiple alignment of Class I chitinases.** Red bar corresponds to a conformational epitope described for the homologous protein hevein [2]. Alignment was produced by Clustal2.1 [1] and shown by using the MView option available at EMBL-EBI [3]

Reference sequence (1): sp|Q05538|CHIC_SOLLC

Identities normalised by aligned length.

Colored by: identity

```
                                   cov     pid   1 [        .         .         .         .         :         .         .         . 80
 1 sp|Q05538|CHIC_SOLLC         100.0% 100.0%      ------------------------------------------------------------MRLSEFTTLFLLFSVLLLSA
 2 sp|P36361|CHI5_PHAVU          97.2%  61.6%      ---------------------------------------------MKK-------NRMMI----MICSVGVVWMLLVGGS
 3 tr|Q42428|Q42428_CASSA        95.0%  66.2%      ----------------------------------------------------------------MKLFSLLLFLAFLLGT
 4 tr|Q6T484|Q6T484_WHEAT        96.0%  69.1%      ------------------------------------------------------------MR--GVVVVAMLAAAFAVSA
 5 tr|P93680|P93680_PERAE        99.1%  68.4%      ----------------------------------------------------------MVYCTASLPLLLLLVGLLAGEA
 6 tr|M0SI55|M0SI55_MUSAM        84.2%  49.9%      MRSRIQHAICRLGHRTSYPKTRLCDFPHSPHLVHRKLHSGQESISLYKHRIPPTPPPPPLLRGMKALLLVIFTLASSLGA
 7 tr|O22318|O22318_MUSAC        35.7%  55.2%      ---------------------------------------------------------------------VIFTLASSLGA
 8 tr|Q8VXF1|Q8VXF1_MUSAC        98.1%  68.8%      ---------------------------------------------------------------MKALLLVIFTLASSLGA
 9 tr|C3VD22|C3VD22_MUSPR        95.3%  70.4%      ---------------------------------------------------------------MKALLLVIFTLASSLGA
10 tr|B6UYK6|B6UYK6_MUSPR        95.7%  68.9%      ---------------------------------------------------------------MKALLLVIFTLASSLGA
11 tr|M0SI56|M0SI56_MUSAM        95.7%  69.5%      ---------------------------------------------------------------MKALLLVIFTLASSLGA
   consensus/100%                                  .....................................................................hhhh.h..hts
   consensus/90%                                   ..................................................................h..hlhhhh..huu
   consensus/80%                                   ................................................................hhhhlhlhhhs.sluA
   consensus/70%                                   ................................................................hhlhlllhshA.uLGA

                                   cov     pid  81          .         1         .         .         .         .         :         . 160
 1 sp|Q05538|CHIC_SOLLC         100.0% 100.0%      SAEQCGSQAGGALCASGLCCSKFGWCGNTNEYCGPGNCQSQCPGGP----------GPSGDLGGVISNSMFDQMLNHRND
 2 sp|P36361|CHI5_PHAVU          97.2%  61.6%      YGEQCGRQAGGALCPGGNCCSQFGWCGSTTDYCGKD-CQSQCGGPS---PA-------PTDLSALISRSTFDQVLKHRND
 3 tr|Q42428|Q42428_CASSA        95.0%  66.2%      SAEQCGRQAGGAACANNLCCSQFGWCGNTAEYCGAG-CQSQCSSPTTTTSSPTASSGGGGDVGSLISASLFDQMLKYRND
 4 tr|Q6T484|Q6T484_WHEAT        96.0%  69.1%      HAEQCGSQAGGATCPNCLCCSKFGFCGTTSDYCGTG-CQSQCNGCSGGTPVPVPTP-SGGGVSSIISQSLFDQMLLHRND
 5 tr|P93680|P93680_PERAE        99.1%  68.4%      FAEQCGRQAGGALCPGGLCCSQFGWCGSTSDYCGPT-CQSQCGGVT---P-----S-PGGGVASLISQSVFNQMLKHRND
 6 tr|M0SI55|M0SI55_MUSAM        84.2%  49.9%      FAEQCGRQAGGALCPGGLCCSQYGWCGNTDPYCGKD-CQSQCGGSG---G------------------------------
 7 tr|O22318|O22318_MUSAC        35.7%  55.2%      FAEQCGRQAGGALCPGGLCCSQYGWCGNTDPYCGQG-CXSQCTGST---PSPSTPS-GGGSVGSIISSSLFEXMLKHRND
 8 tr|Q8VXF1|Q8VXF1_MUSAC        98.1%  68.8%      FAEQCGRQAGGALCPGGLCCSQYGWCGNTDPYCGQG-CQSQCGGSG---G-----S-GGGSVASIISSSLFEQMLKHRND
 9 tr|C3VD22|C3VD22_MUSPR        95.3%  70.4%      FAEQCGRQAGGALCPGGLCCSQYGWCGNTDPYCGEG-CQSQCGGS------------SGGSVASIISSSLFEQMLKHRND
10 tr|B6UYK6|B6UYK6_MUSPR        95.7%  68.9%      FAEQCGRQAGGALCPGGLCCSQYGWCGNTDPYCGQG-CQSQCTGST---PSPSTPS-GGGSVGSIISSSLFEQMLKHRND
11 tr|M0SI56|M0SI56_MUSAM        95.7%  69.5%      FAEQCGRQAGGALCPGGLCCSQYGWCGNTDPYCGQG-CQSQCTGST---PSPSTPS-GGGSVGSIISSSLFEQMLKHRND
   consensus/100%                                  .uEQCGpQAGGAhCsss.CCSpaGaCGsTs.YCG.s.C.SQCsus...................................
   consensus/90%                                   .AEQCGpQAGGAhCsssLCCSpaGWCGsTs.YCG.s.CQSQCsGss............sssluulIStShFp.hL.aRND
   consensus/80%                                   aAEQCGRQAGGALCPuGLCCSQaGWCGsTssYCGts.CQSQCsGss...s.......suGsluulISpShF-QMLpHRND
   consensus/70%                                   aAEQCGRQAGGALCPGGLCCSQaGWCGNTssYCGpG.CQSQCsGss...s.......sGGsVuSlISpSlF-QMLKHRND

                                   cov     pid 161          .         .         .         2         .         .         .         . 240
 1 sp|Q05538|CHIC_SOLLC         100.0% 100.0%      NACQGKNNFYSYNAFVTAAGSFPGFGTTGDITARKREIAAFLAQTSHETTGGWPTAPDGPYAWGYCFLREQGSPGDYCTP
 2 sp|P36361|CHI5_PHAVU          97.2%  61.6%      GACPAK-GFYTYDAFIAAAKAYPSFGNTGDTATRKREIAAFLGQTSHETTGGWATAPDGPYAWGYCFVRERNP-SAYCSA
 3 tr|Q42428|Q42428_CASSA        95.0%  66.2%      PRCKSN-GFYTYNAFIAAARSFNGFGTTGDVTTRKRELAAFLAQTSHETTGGWATAPDGPYAWGYCFVMENNK-QTYCT-
 4 tr|Q6T484|Q6T484_WHEAT        96.0%  69.1%      AACLAK-GFYNYGAFVAAANSFSGFATTGSTDVKKREVAAFLAQTSHETTGGWPTAPDGPYSWGYCFNQERGATSDYCTP
 5 tr|P93680|P93680_PERAE        99.1%  68.4%      AACQAK-GFYTYNAFIAAANSFNGFASVGDTATRKREIAAFLAQTSHETTGGWATAPDGPYAWGYCFLKEQGNPPDYCVP
 6 tr|M0SI55|M0SI55_MUSAM        84.2%  49.9%      ----GS-GFYTYTAFIAAANFFSGFGTTGDDTARKREIAAFLAQTSHETTGGWATAPDGPYAWGYCFVQEQNPPSDYCVA
 7 tr|O22318|O22318_MUSAC        35.7%  55.2%      AACPGK-GFYTYNAFIAAANSFSGFGTTGDXATXXRE-------------------------------------------
 8 tr|Q8VXF1|Q8VXF1_MUSAC        98.1%  68.8%      AACPGK-GFYTYNAFIAAANSFSGFGTTGDDAKKKREIAAFLAQTSHETTGGWATAPDGPYAWGYCFVQEQNPSSDYCVA
```

```
 9 tr|C3VD22|C3VD22_MUSPR  95.3%  70.4%   AACPGK-GFYTYNAFIAAASSFSGFGTTGDDATKKREIAAFLAQTSHETTGGWATAPDGPYAWGYCFVQEQNPSSDYCVA
10 tr|B6UYK6|B6UYK6_MUSPR  95.7%  68.9%   AACPGN-GFYTYNAFIAAANSFSGFGTTGDDATKKREIAAFLAQTSHETTGGWATAPDGPYAWGYCFVQEQNPSSDYCVA
11 tr|M0SI56|M0SI56_MUSAM  95.7%  69.5%   AACPGK-GFYMYNAFIAAANSFSGFGTTGDDATKKREIAAFLAQTSHETTGGWATAPDGPYAWGYCFVQEQNPPSDYCVA
   consensus/100%                        ....up.sFY.YsAFlsAAt.asuFussGs.sh..RE...........................................
   consensus/90%                         stC.up.GFYsYsAFlAAApuFsGFuoTGD.ss+KRElAAFLuQTSHETTGGWsTAPDGPYuWGYCF..Eps...sYCs.
   consensus/80%                         sAC.up.GFYoYsAFIAAAsSFsGFGTTGDsss+KRElAAFLAQTSHETTGGWsTAPDGPYAWGYCFlpEpss.ssYCss
   consensus/70%                         uAC.uK.GFYTYNAFIAAAsSFsGFGTTGDsss+KREIAAFLAQTSHETTGGWATAPDGPYAWGYCFlpEpsssuDYCss

                             cov    pid 241          :         .         .         .         .         3         .         . 320
 1 sp|Q05538|CHIC_SOLLC  100.0% 100.0%   SSQWPCAPGRKYFGRGPIQISHNYNYGPCGRAIGVDLLNNPDLVATDPVISFKSAIWFWMTPQSPKPSCHDVITGRWQPS
 2 sp|P36361|CHI5_PHAVU    97.2%  61.6%   TPQFPCAPGQQYYGRGPIQISWNYNYGQCGRAIGVDLLNKPDLVATDSVISFKSALWFWMTAQSPKPSSHDVITSRWTPS
 3 tr|Q42428|Q42428_CASSA  95.0%  66.2%   SKSWPCVFGKQYYGRGPIQLTHNYNYGQAGKAIGADLINNPDLVATNPTISFKTAIWFWMTPQANKPSSHDVIIGNWRPS
 4 tr|Q6T484|Q6T484_WHEAT  96.0%  69.1%   SSQWPCAPGKKYFGRGPIQISHNYNYGPAGQAIGTDLLNNPDLVASDATVSFKTALWFWMTPQSPKPSSHDVITGRWSPS
 5 tr|P93680|P93680_PERAE  99.1%  68.4%   TAQWPCAPGKKYYGRGPIQISYNYNYGPAGRAIGYDLINNPDAVATDPVISFKTALWFWMTPQSPKPSCHNVITGRWTPS
 6 tr|M0SI55|M0SI55_MUSAM  84.2%  49.9%   SSQWPCAAGKKYYGRGPIQISFNYNYGPAGRAIGSDLLNNPDLVATDATISFKTALWFWMTPQSPKP-------------
 7 tr|O22318|O22318_MUSAC  35.7%  55.2%   --------------------------------------------------------------------------------
 8 tr|Q8VXF1|Q8VXF1_MUSAC  98.1%  68.8%   SSQWPCAAGKKYYGRGPIQISFNYNYGPAGRAIGSDLLNNPDLVATDATISFKTALWFWMTPQSPKPSCHDVITGSWTPS
 9 tr|C3VD22|C3VD22_MUSPR  95.3%  70.4%   SSQWPCAAGKKYYGRGPIQISFNYNYGPAGRAIGSDLLNNPDLVATDATISFKTALWFWMTPQSPKPSCHNVITGGWTPS
10 tr|B6UYK6|B6UYK6_MUSPR  95.7%  68.9%   SSQWPCAAGKKYYGRGPIQISFNYNYGPAGRAIGSDLLNNPDLVATDATISFKTALWFWMTPQSPKPSCHNVITGGWTPS
11 tr|M0SI56|M0SI56_MUSAM  95.7%  69.5%   SSQWPCAAGKKYYGRGPIQISFNYNYGPAGRAIGSDLLNNPDLVATDATISFKTALWFWMTPQSPKPSCHDVITGSWTPS
   consensus/100%                        ................................................................................
   consensus/90%                         o.paPCs.GppYaGRGPIQloaNYNYG.sGpAIG.DLlNpPDhVAossslSFKoAlWFWMTsQusKP.............
   consensus/80%                         osQWPCAsG+pYaGRGPIQISaNYNYG.sG+AIGsDLlNNPDLVATDssISFKoAlWFWMTPQSPKPSsHsVIhutWpPS
   consensus/70%                         SuQWPCAsGKKYYGRGPIQISaNYNYGPAGRAIGsDLLNNPDLVATDssISFKTALWFWMTPQSPKPSsHsVITGtWpPS

                             cov    pid 321          .         .         :         .         .         .         .    ] 396
 1 sp|Q05538|CHIC_SOLLC  100.0% 100.0%   GADQAANRVPGFGVITNIINGGLECGHGSDSRVQDRIGFYRRYCGILGVSPGENLDCGNQRSFGNGLLVDIM----
 2 sp|P36361|CHI5_PHAVU    97.2%  61.6%   SADVAARRLPGYGTVTNIINGGLECGRGQDSRVQDRIGFFKRYCDLLGVGYGNNLDCYSQTPFGNSLFLSDLVTSQ
 3 tr|Q42428|Q42428_CASSA  95.0%  66.2%   AADTSAGRVPSYGVITNIINGGLECGHGSDDRVANRIGFYKRYCDTLGVSYGNNLDCYNQKPFA------------
 4 tr|Q6T484|Q6T484_WHEAT  96.0%  69.1%   GADQAAGRVPGYGVITNIINGGLECGRGQDARVADRIGFYKRYCDLLGVSYGDNLDCYNQRPFA------------
 5 tr|P93680|P93680_PERAE  99.1%  68.4%   AADRAAGRLPGYGVITNIINGGIECGKGFNDKVADRIGFYKRYCDLLGVSYGSNLDCYNQRSFGVSTNPLAASS--
 6 tr|M0SI55|M0SI55_MUSAM  84.2%  49.9%   --------RPGYGVTTNIINGGLECGKGYDARVADRIGFYKRYCDLLGVSYGDNLDCYNQRPFASTAATATF----
 7 tr|O22318|O22318_MUSAC  35.7%  55.2%   ----------------------------------------------------------------------------
 8 tr|Q8VXF1|Q8VXF1_MUSAC  98.1%  68.8%   NADQAAGRLPGYGVTTNIINGGLECGKGYDARVADRIGFYKRYCDLLGVSYGDNLDCYNQRPFASTAATATF----
 9 tr|C3VD22|C3VD22_MUSPR  95.3%  70.4%   NADRAAGRLPGYGVTTNIINGGLECGKGSDARVADRIGFYKRYCDLLGVSYGDNLDCYNQSPFT------------
10 tr|B6UYK6|B6UYK6_MUSPR  95.7%  68.9%   NADRAAGRLPGYGVTTNIINGGLECGKGSDARVADRIGFYKRYCDLLGVSYGDNLDCYNQSPFT------------
11 tr|M0SI56|M0SI56_MUSAM  95.7%  69.5%   NADRAAGRLPGYGVTTNIINGGLECGKGSDARVADRIGFYKRYCDLLGVSYGDNLDCYSQSPFT------------
   consensus/100%                        ............................................................................
   consensus/90%                         ........hPuaGshTNIINGGlECG+G.ss+VtsRIGFa+RYCshLGVu.GpNLDChsQpsFs............
   consensus/80%                         sAD.uAtRlPGYGVhTNIINGGLECG+G.DsRVtDRIGFYKRYCDlLGVSYGsNLDCYsQpsFs............
   consensus/70%                         sADpAAsRlPGYGVhTNIINGGLECG+G.DuRVADRIGFYKRYCDLLGVSYGsNLDCYNQpPFs............
```

**Figure S2. Multiple alignment of Class II chitinases**. Red bars correspond to conformational epitopes described for the rice chitinase [4]. Alignment was produced by Clustal2.1 [1] and shown by using the MView option available at EMBL-EBI [3].

Reference sequence (1): tr|Q7Y0S1|Q7Y0S1_SOLLC

Identities normalised by aligned length. Colored by: identity

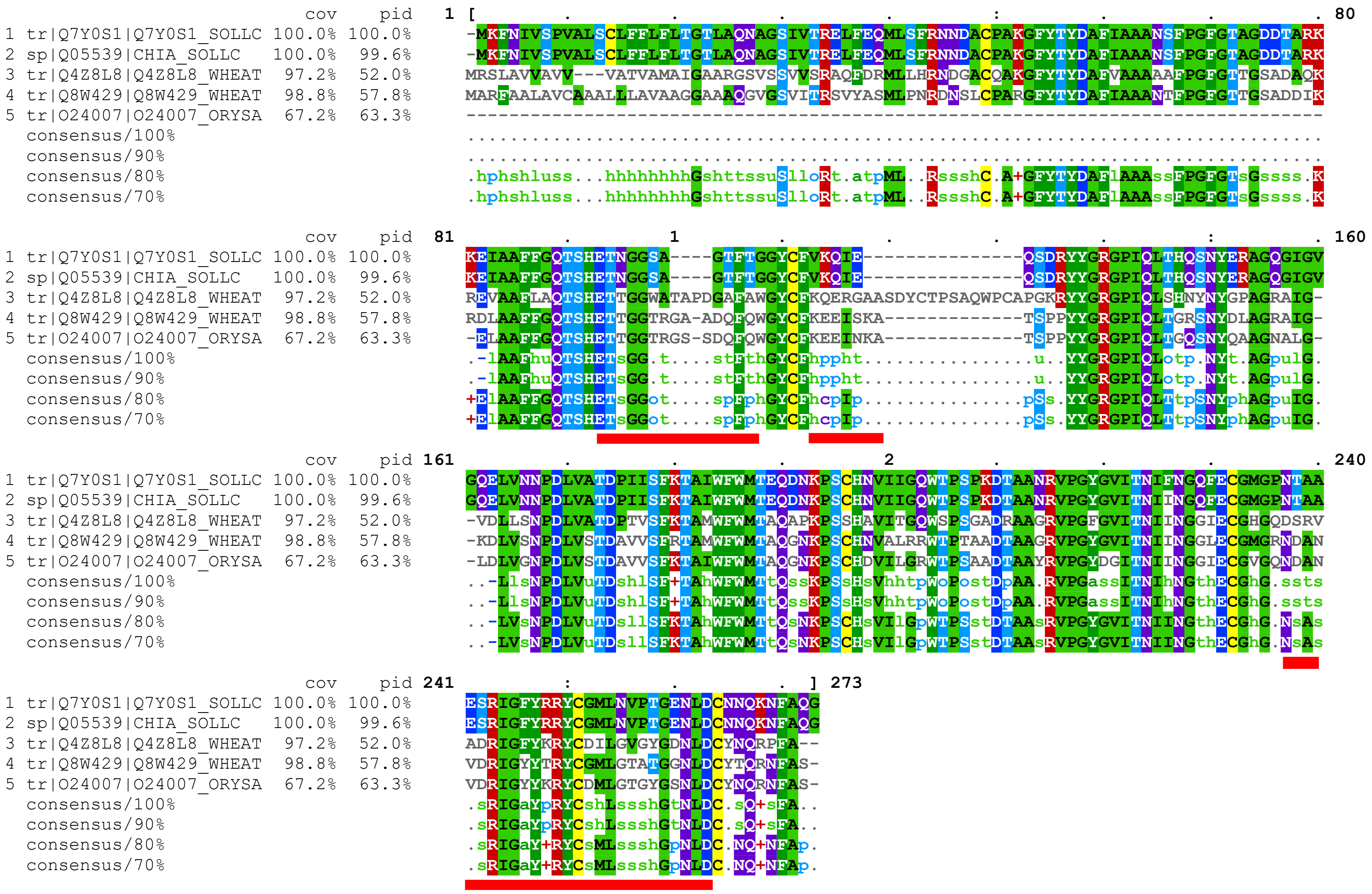

**Figure S3. Multiple alignment of Class III chitinases**. Red bar corresponds to a conformational epitope described for the pomegranate chitinase [5]. Alignment was produced by Clustal2.1 [1] and shown by using the MView option available at EMBL-EBI [3].

Reference sequence (1): tr|D7REL9|D7REL9_COFAR

Identities normalised by aligned length. Colored by: identity

```
                                  cov     pid   1 [         .         .         .         .         :         .         .         . 80
1 tr|D7REL9|D7REL9_COFAR 100.0% 100.0%     ----------------------------AGIVRYWGRGHD-EPSSLAEFGRQEFATDVNIAFLEDFGGGRMPELNISHPL
2 tr|Q2VST0|Q2VST0_9ROSA 100.0%  27.0%     ---MVPQAKLVVASLILTSALIQTSEAVGGIATYWGQYTETEEGSLAEACASNLYSYINIAYLNIFGEGRYLSLNISGHC
3 tr|G1UH28|G1UH28_PUNGR  95.8%  30.4%     MAKTLPFSRALLL--SLSILLVARAISAGDIAIYWGQN--GGEGTLASTCDTGRYAYVIVSFVTTFGNFRAPVVNLAGHC
  consensus/100%                           ............................usIshYWGp....t.uoLAphstpthhs.l.lualp.FGthRh..lNlut.h
  consensus/90%                            ............................usIshYWGp....t.uoLAphstpthhs.l.lualp.FGthRh..lNlut.h
  consensus/80%                            ............................usIshYWGp....t.uoLAphstpthhs.l.lualp.FGthRh..lNlut.h
  consensus/70%                            ............................usIshYWGp....t.uoLAphstpthhs.l.lualp.FGthRh..lNlut.h

                                  cov     pid  81           .         1         .         .         .         .         :         . 160
1 tr|D7REL9|D7REL9_COFAR 100.0% 100.0%     P--------SASDIEYCQKHQTKVFISIAGQ---PSLSSVEDAEEVAAYVWNTYLGGKSSD----RPFGKAVLDGVELHI
2 tr|Q2VST0|Q2VST0_9ROSA 100.0%  27.0%     S----DCTFLGEEIKACQSQGVKIFLSLGGPYGDYHLTTDGDADRVAEQLWSSFLGGSKSTGVYQPLLGDVELDGIDLDI
3 tr|G1UH28|G1UH28_PUNGR  95.8%  30.4%     DPAAGTCTGLSDEIRSCQGKDIKVLMSIGGGAGDYSLVSEADADNFADYLWNNFLGGQSSS----RPLGDAVLDGIDFDI
  consensus/100%                           s.........up-Ic.CQtpthKlhhSluG.....pLso.tDA-phAt.lWssaLGGppSs......hGcs.LDGl-hcI
  consensus/90%                            s.........up-Ic.CQtpthKlhhSluG.....pLso.tDA-phAt.lWssaLGGppSs......hGcs.LDGl-hcI
  consensus/80%                            s.........up-Ic.CQtpthKlhhSluG.....pLso.tDA-phAt.lWssaLGGppSs......hGcs.LDGl-hcI
  consensus/70%                            s.........up-Ic.CQtpthKlhhSluG.....pLso.tDA-phAt.lWssaLGGppSs......hGcs.LDGl-hcI

                                  cov     pid 161           .         .         .         2         .         .         .         . 240
1 tr|D7REL9|D7REL9_COFAR 100.0% 100.0%     HSGNTTYLDDLARALKGYSNV-----ISAVAAECPIP---DPALDTTIRTGVVDQVRVEFFDNPS--CQFTPPK--DTSL
2 tr|Q2VST0|Q2VST0_9ROSA 100.0%  27.0%     QIGPPEEYDVLARNLKDLTKDRTRPFYLSAAPKCSAYNDSDAYLWTAVETGLFDFVWVKFYNDTS--CQYNNDTAAGLDA
3 tr|G1UH28|G1UH28_PUNGR  95.8%  30.4%     ELGTTTFYDTLARALSSRSTQ-AAKVYLTAAPQCPHP---DSHLDAALNTGLFDNVWIQFYNNPLAQCQYSSGN---TND
  consensus/100%                           p.Gssp.hDsLARsLpshop......h.ssAspCsh....DshL.sslpTGlhD.VhlpFasss...CQasssp...hs.
  consensus/90%                            p.Gssp.hDsLARsLpshop......h.ssAspCsh....DshL.sslpTGlhD.VhlpFasss...CQasssp...hs.
  consensus/80%                            p.Gssp.hDsLARsLpshop......h.ssAspCsh....DshL.sslpTGlhD.VhlpFasss...CQasssp...hs.
  consensus/70%                            p.Gssp.hDsLARsLpshop......h.ssAspCsh....DshL.sslpTGlhD.VhlpFasss...CQasssp...hs.

                                  cov     pid 241           :         .         .         .         .         3         .         . 320
1 tr|D7REL9|D7REL9_COFAR 100.0% 100.0%     LFPSWDNWSDYPG-VHKLYLGIPISPTI--APEGGYIPPNELVYHVLPYLKKSPVYGGIMVFPYLHHE-VNFQS-MLRSY
2 tr|Q2VST0|Q2VST0_9ROSA 100.0%  27.0%     FYRSWYDWTVSLAEGNKLLIGIPASNETDNSPLGGYIPSDVLNDQIVSVIMTSSKFGGVNVWNRYYDLKTNYSSSIILEY
3 tr|G1UH28|G1UH28_PUNGR  95.8%  30.4%     ILSSWNTWTSSTTAG-KIFLGLPAAPEA---AGSGYIPPDVLTGQILPQIKTSAKYGGVMLYSKFYDTTYST--TIKDQV
  consensus/100%                           hh.SW.sWos..s.s.KlhlGlPhusph...s.uGYIPss.Ls.plls.lhpSshaGGl.lashhac..hsh...hh.ph
  consensus/90%                            hh.SW.sWos..s.s.KlhlGlPhusph...s.uGYIPss.Ls.plls.lhpSshaGGl.lashhac..hsh...hh.ph
  consensus/80%                            hh.SW.sWos..s.s.KlhlGlPhusph...s.uGYIPss.Ls.plls.lhpSshaGGl.lashhac..hsh...hh.ph
  consensus/70%                            hh.SW.sWos..s.s.KlhlGlPhusph...s.uGYIPss.Ls.plls.lhpSshaGGl.lashhac..hsh...hh.ph

                                  cov     pid 321           .         ] 339
1 tr|D7REL9|D7REL9_COFAR 100.0% 100.0%     ARAA---------------
2 tr|Q2VST0|Q2VST0_9ROSA 100.0%  27.0%     VNSGTKYLPLRTKFMYQNA
3 tr|G1UH28|G1UH28_PUNGR  95.8%  30.4%     -------------------
  consensus/100%                           ...................
  consensus/90%                            ...................
  consensus/80%                            ...................
  consensus/70%                            ...................
```

**Figure S4. Multiple alignment of Class IV chitinases**. Red bars correspond to a conformational epitope described for the rice chitinase [4]. Alignment was produced by Clustal Omega [1] and shown by using the MView option available at EMBL-EBI [3]. Reference sequence (1): sp|P29022.1|CHIA_MAIZE. Identities normalised by aligned length. Colored by: identity.

```
                                 cov     pid   1 [        .         .         .         .         :         .         .         . 80
1 sp|P29022.1|CHIA_MAIZE 100.0% 100.0%     MANAPRILALGLLALLCAAAGPAAAQNCGCQPNFCCSKFGYCGTTDAYCGDGCQSGPCRSGGGGGGGGGGGGGGSGGANV
2 U97522                  92.1%  63.9%     -------L---TVLLVGALFGAAVAQNCGCASGLCCSKYGYRGTGSDYCGDGCQSGPCDSGSGS------------GSSV
3 AAQ10093.1              93.9%  63.1%     --MAAKLL---TVLLVGALFGAAVAQNCGCASGLCCSKYGYCGTGSDYCGDGCQSGPCDSSSGS------------GSSV
4 XP_002275386.1          93.9%  63.1%     --MAAKLL---TVLLVGALFGAAVAQNCGCASGLCCSKYGYCGTGSDYCGDGCQSGPCDSSSGS------------GSSV
5 AAB65776.1              92.9%  62.0%     --MAAKLL---TVLLVGALFGAAVAQNCGCASGLCCSKYGYCGTGSDYCGDGCQSGPCDSS--------------GSSV
  consensus/100%                           .......L...hlhLlsAhhGsAsAQNCGCtsshCCSKaGYpGTsssYCGDGCQSGPCcSu...............GusV
  consensus/90%                            .......L...hlhLlsAhhGsAsAQNCGCtsshCCSKaGYpGTsssYCGDGCQSGPCcSu...............GusV
  consensus/80%                            ...As+lL...TVLLVGALFGAAVAQNCGCASGLCCSKYGYCGTGSDYCGDGCQSGPCDSuuGu............GSSV
  consensus/70%                            ...As+lL...TVLLVGALFGAAVAQNCGCASGLCCSKYGYCGTGSDYCGDGCQSGPCDSuuGu............GSSV

                                 cov     pid  81          .         1         .         .         .         .         :         . 160
1 sp|P29022.1|CHIA_MAIZE 100.0% 100.0%     ANVVTDAFFNGIKNQAGSGCEGKNFYTRSAFLSAVNAYPGFAHGGTEVEGKREIAAFFAHVTHETGHFCYISEINK-SNA
2 U97522                  92.1%  63.9%     SDIVTQAFFDGIINQAASSCAGKNFYTRAAFLSALNSYSGFGNDGSTDANKREIAAFFAHVTHETGHFCYIEEINGASHN
3 AAQ10093.1              93.9%  63.1%     SDIVTQSFFDGIINQAASSCAGKNFYTRAAFLSALNSYSGFGNDGSTDANKREIAAFFAHVTHETGHFCYIEEINGASHN
4 XP_002275386.1          93.9%  63.1%     SDIVTQSFFDGIINQAASSCAGKNFYTRAAFLSALNSYSGFGNDGSTDANKREIAAFFAHVTHETGHFCYIEEINGASHN
5 AAB65776.1              92.9%  62.0%     SDIVTQSFFDGIISQAASSCAGKNFYTRAAFLSALNSYSGFGNDGSTDANKREIAAFFAHVTHETGHFCYIEEINGASHN
  consensus/100%                           uslVTpuFFsGIhsQAuSuCtGKNFYTRuAFLSAlNuYsGFupsGopstsKREIAAFFAHVTHETGHFCYIpEINt.Sps
  consensus/90%                            uslVTpuFFsGIhsQAuSuCtGKNFYTRuAFLSAlNuYsGFupsGopstsKREIAAFFAHVTHETGHFCYIpEINt.Sps
  consensus/80%                            SDIVTQuFFDGIINQAASSCAGKNFYTRAAFLSALNSYSGFGNDGSTDANKREIAAFFAHVTHETGHFCYIEEINGASHN
  consensus/70%                            SDIVTQuFFDGIINQAASSCAGKNFYTRAAFLSALNSYSGFGNDGSTDANKREIAAFFAHVTHETGHFCYIEEINGASHN

                                cov     pid 161         .         .         .         2         .         .         .         . 240
1 sp|P29022.1|CHIA_MAIZE 100.0% 100.0%     YCDASNRQWPCAAGQKYYGRGPLQISWNYNYGPAGRDIGFNGLADPNRVAQDAVIAFKTALWFWMNNVHGVMPQGFGATI
2 U97522                  92.1%  63.9%     YCDSSNTQYPCVSGQNYYGRGPLQLTWNYNYGAAGNSIGFNGLSNPGIVATDVVTSFKTALWFWMNNVHSVLSQGFGATI
3 AAQ10093.1              93.9%  63.1%     YCDSSNTQYPCVSGQNYYGRGPLQLTWNYNYGAAGNSIGFNGLSNPGIVATDVVTSFKTALWFWMNNVHSVIGQGFGATI
4 XP_002275386.1          93.9%  63.1%     YCDSSNTQYPCVSGQNYYGRGPLQLTWNYNYGAAGNSIGFNGLSNPGIVATDVVTSFKTALWFWMNNVHSVIGQGFGATI
5 AAB65776.1              92.9%  62.0%     YCDSSNTQYPCVSGQNYYGRGPLQLTWNYNYGAAGNSIGFNGLSNPGIVATDVVTSFKTALWFWMNNVHSVIGQGFGATI
  consensus/100%                           YCDuSNpQaPCsuGQpYYGRGPLQloWNYNYGsAGpsIGFNGLusPshVApDsVhuFKTALWFWMNNVHuVhsQGFGATI
  consensus/90%                            YCDuSNpQaPCsuGQpYYGRGPLQloWNYNYGsAGpsIGFNGLusPshVApDsVhuFKTALWFWMNNVHuVhsQGFGATI
  consensus/80%                            YCDSSNTQYPCVSGQNYYGRGPLQLTWNYNYGAAGNSIGFNGLSNPGIVATDVVTSFKTALWFWMNNVHSVluQGFGATI
  consensus/70%                            YCDSSNTQYPCVSGQNYYGRGPLQLTWNYNYGAAGNSIGFNGLSNPGIVATDVVTSFKTALWFWMNNVHSVluQGFGATI

                                 cov     pid 241          :         .         .        .] 281
1 sp|P29022.1|CHIA_MAIZE 100.0% 100.0%     RAINGALECNGNNPAQMNARVGYYKQYCQQLRVDPGPNLIC
2 U97522                  92.1%  63.9%     QAINGAVECNGGNTAAVNARVQYYKDYCSQLGVSPGDNLTC
3 AAQ10093.1              93.9%  63.1%     RAINGAVECNGGNTAAVNARVQYYKDYCSQLGVSPGDNLTC
4 XP_002275386.1          93.9%  63.1%     RAINGAVECNGGNTAAVNARVQYYKDYCSQLGVSPGDNLTC
5 AAB65776.1              92.9%  62.0%     RAINGAVECNGGNTAAVNARVQYYKDYCSQLGASPGDNLTC
  consensus/100%                           pAINGAlECNGsNsAthNARVtYYKpYCpQLtssPGsNLhC
  consensus/90%                            pAINGAlECNGsNsAthNARVtYYKpYCpQLtssPGsNLhC
  consensus/80%                            RAINGAVECNGGNTAAVNARVQYYKDYCSQLGVSPGDNLTC
  consensus/70%                            RAINGAVECNGGNTAAVNARVQYYKDYCSQLGVSPGDNLTC
```

**References**


1. Sievers, F.; Wilm, A.; Dineen, D.; Gibson, T.J.; Karplus, K.; Li, W.; Lopez, R.; McWilliam, H.; Remmert, M.; Söding, J.; et al. Fast, scalable generation of high-quality protein multiple sequence alignments using Clustal Omega. *Mol. Syst. Biol.* **2011**, *7*, 539.
2. Alenius, H.; Kalkkinen, N.; Reunala, T.; Turjanmaa, K.; Palosuo, T. The main IgE-binding epitope of a major latex allergen, prohevein, is present in its N-terminal 43-amino acid fragment, hevein. *J. Immunol.* **1996**, *156*, 1618–1625.
3. Chojnacki, S.; Cowley, A.; Lee, J.; Foix, A.; Lopez, R. Programmatic access to bioinformatics tools from EMBL-EBI update: 2017. *Nucleic Acids Research* **2017**, *45*, W550–W553.
4. Mishra, A.; Gaur, S.N.; Lavasa, S.; Arora, N. In vitro assessment of allergenicity features and localization of probable IgE binding regions. *Food and Chemical Toxicology* **2015**, *84*, 181–187.
5. Tuppo, L.; Giangrieco, I.; Alessandri, C.; Ricciardi, T.; Rafaiani, C.; Ciancamerla, M.; Ferrara, R.; Zennaro, D.; Bernardi, M.L.; Tamburrini, M.; et al. Pomegranate chitinase III: Identification of a new allergen and analysis of sensitization patterns to chitinases. *Mol. Immunol.* **2018**, *103*, 89–95.
